# Epstein–Barr virus-associated gastric adenosquamous carcinoma with concurrent gastric carcinoma with lymphoid stroma: a case report and review of the literature

**DOI:** 10.1186/s12876-022-02417-4

**Published:** 2022-07-16

**Authors:** Fang Cao, Yan Yan, Dongfeng Niu, Xiaozheng Huang, Ling Jia, Xinting Diao, Zhongwu Li

**Affiliations:** 1 Key Laboratory of Carcinogenesis and Translational Research (Ministry of Education), Department of Pathology, Peking University Cancer Hospital & Institute, 52 Fucheng Road, HaiDian District, Beijing, 100142 China; 2 Key Laboratory of Carcinogenesis and Translational Research (Ministry of Education), Department of Endoscopy, Peking University Cancer Hospital & Institute, Beijing, China

**Keywords:** Gastric cancer, Adenosquamous carcinoma, Gastric carcinoma with lymphoid stroma, Epstein–Barr virus

## Abstract

**Background:**

Adenosquamous carcinoma (ASC)with concurrent gastric carcinoma with lymphoid stroma (GCLS) are extremely rare tumors. There are only limited cases reported in the literature. Epstein–Barr virus (EBV) infection was found in the concomitant GCLS, but none in the ASC. Here, we report the first case of gastric cancer with EBV infection detected in both ASC and GCLS.

**Case presentation:**

A 59-year-old man complained of intermittent upper abdominal pain. The gastric endoscopy revealed a type IIc tumor located in the gastric body near the fundus of the stomach. Histological examination of the gastric tumor showed the coexistence of ASC and GCLS. Both components were positive for EBV-encoded RNA (EBER) in situ hybridization. Neoplastic nests of the former were positive for p63, p40 and CK5/6. The glandular components showed positive acid mucus in the Alcian-blue periodic-acid-schiff (AB-PAS) staining. There was significant difference in the expression of epidermal growth factor receptor (EGFR) between adenocarcinoma and squamous carcinoma, but not in other proteins such as human epidermal growth factor receptor 2 (HER2), p53 and mismatch repair proteins. The role of EGFR signaling pathway needs to be further explored in the differentiation of squamous carcinoma in the gastric ASC. Finally, a diagnosis of early EBV associated gastric ASC with concurrent GCLS (pT1bN1) was made. The patient took a single-drug S1 periodically for half a year after the surgery and has been disease free during 8 months of medical follow-up.

**Conclusions:**

This is the first case of EBV associated gastric ASC with concurrent GCLS, and pathologists and clinicians should recognize and pay attention to this type of tumor.

## Background

Epstein–Barr virus (EBV) associated gastric cancer (EBV-GC) accounts for about 10% of gastric cancer. This cancer type has received more and more attention recently due to its unique genetic and epigenetic features, which could be potential targets for cancer treatment [1]. EBV-GC is almost all adenocarcinoma, and a special histopathological variant is gastric carcinoma with lymphoid stroma (GCLS), which has abundant lymphocytes infiltrating within and around the tumor cells. Patients with GCLS have a relatively better prognosis than those with conventional gastric adenocarcinoma [2].

Primary gastric adenosquamous carcinoma (ASC) is rare and aggressive, which accounts for less than 1% in gastric carcinomas [3]. Gastric ASC is a tumor in which the components of adenocarcinoma (AC) and squamous cell carcinoma (SCC) mix in different proportions. According to the classification of digestive tumors introduced by the World Health Organization (WHO), ASC is diagnosed when the SCC component exceeds 25% of the primary tumor [4]. Gastric ASC has distinct features and a poorer prognosis. Its clinical management is still under constant discussion [5].

In recent years, there have been a small number of reports on gastric ASC with concurrent GCLS. The biological behavior of this rare tumor is extremely difficult to predict. EBV infection was believed to play an important role in the development of GCLS components, since EBV infection has been frequently detected in these components, but much less often in the ASC components. Only one report in the literature revealed that EBV infection could be focally detected in the SCC components of gastric GCLS with focal SCC differentiation [6]. The role of EBV in the carcinogenesis of ASC remains unclear. In this article, we reported the first case of gastric ASC with concurrent GCLS, with evidence of EBV infection found in both ASC and GCLS, and summarized the clinicopathological features of those previous related cases, as compared with the present one.

## Case presentation

A 59-year-old Chinese man complained of intermittent upper abdominal pain for 1 month, which worsened when he was hungry, and relieved after eating, accompanied by intermittent bowel movements, and no discomfort such as nausea or vomiting. The patient was in good health before, and the special family history of tumor was that his younger brother had liver cancer. Serum tumor biomarker examination revealed his CA125 increased to 66.3u/ml, and the normal level was 0-35.2 u/ml. Gastric laparoscopic examination revealed a type-IIc tumor in the gastric body with a diameter of 3 cm, and biopsy of the lesion revealed signet ring cell carcinoma. Subsequently, the patient underwent total gastrectomy with D2 lymph node dissection.

Routine histological examination was conducted and formalin-fixed and paraffin-embedded full-thickness specimens of the tumor were collected and stained with hematoxylin and eosin (HE stain). Primary antibodies against p40 (2R-8), p53 (Do-7), MLH1 (GM002), MSH2 (RED2), MSH6 (EP49), PMS2 (EP51) (Gene Tech, Shanghai, China), p63 (UMAB4/4A4), EGFR (EP22), CK7 (EP16) ,CK5/6 (OTI1C7) (ZSGB-Bio, Beijing, China),and HER2 (VENTANA anti-HER2/neu 4B5, Roche, Basel, Switzerland) were purchased as ready-made working solution, and immunohistochemical (IHC) staining was performed on autostainer (BOND-III, Leica Biosystems, Ltd., Newcastle, UK) or VENTANA benchmark ultra (Roche Diagnostics, USA) with specified second antibody and visualization system. AB-PAS staining was performed according to the manufacturer’s instructions (BenchMark Special Stains, Roche Diagnostics, USA). For detection of EBV-encoded RNA, the EBER 1 Probe was used (Leica, Newcastle, UK). In situ hybridization analysis was performed using autostainers (BOND-III, Leica Biosystems, Ltd., Newcastle, UK). The expression of programmed death ligand 1 (PD-L1) was analyzed using the PD-L1 antibody (clone 22C3) on the Dako Automated Link 48 platform (Dako, Carpenteria, CA) with proper controls. All sections were observed through LEICA DM3000 LED (Leica Microsystems, UK). Electronic slices were obtained using P250 FLASH Scanning System (3D HISTECH, Ltd, China) and characteristic images were captured by the CaseViewer software (3D HISTECH, Ltd, China).

**Fig. 1:**
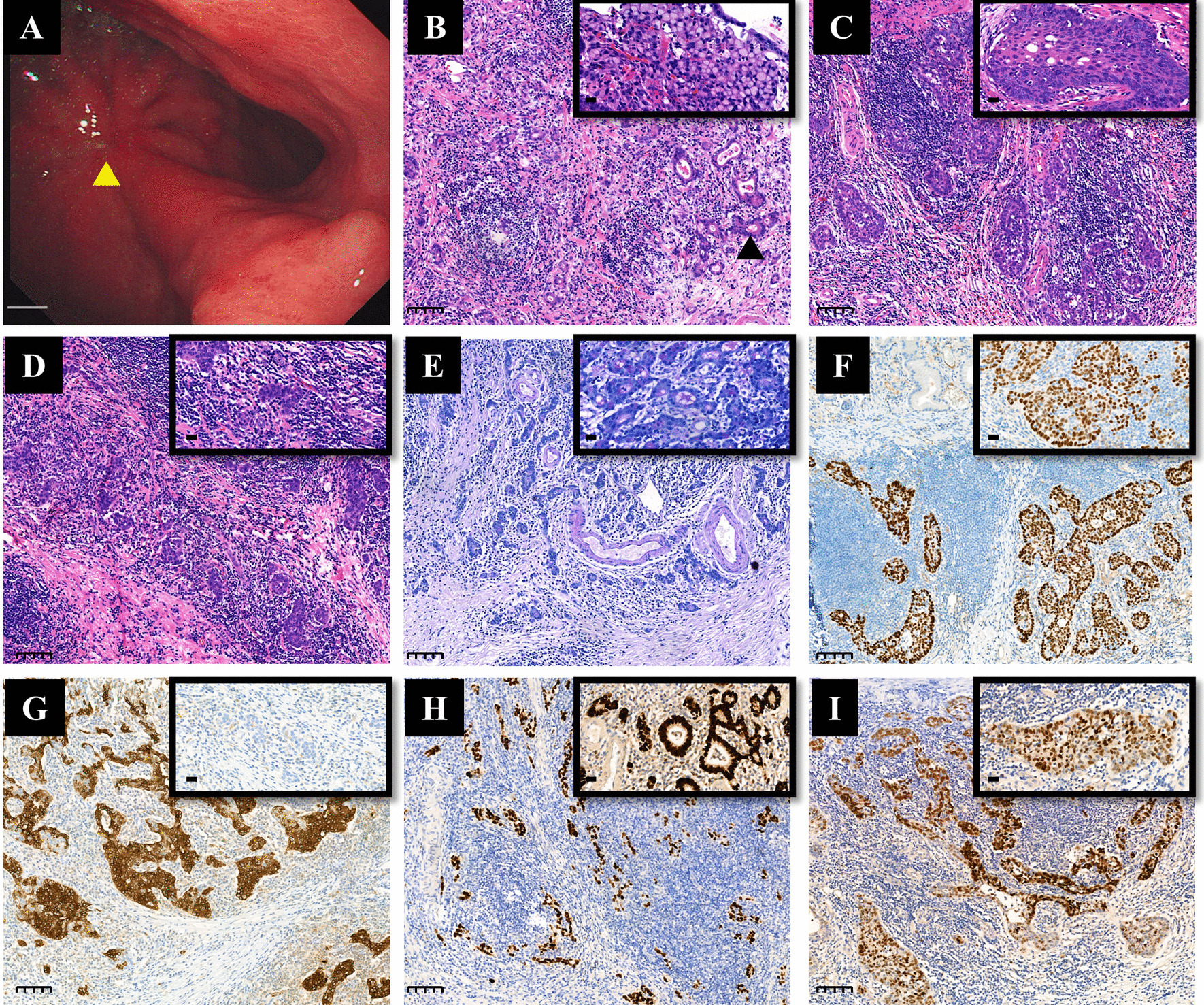
Endoscopic, histopathological, immunohistochemical and in situ hybridization findings of the gastric tumors. **A** Endoscopic findings. The type IIc tumor is located in the gastric body near the fundus of the stomach (*Triangle*). The scale bar represents 1 cm. **B** Histopathological features of the AC component (accounting for 30%). Poorly differentiated tubular AC with focal glandular formation (*Triangle*, H&E; magnification, ×100, scale bar: 100 μm) and signet ring cell carcinoma (inset; H&E; magnification, ×400, scale bar: 20 μm). **C** Histopathological features of the SCC component (accounting for 40%). SCC component shows solid nest or trabecular proliferation of tumor cells with relatively rich eosinophilic cytoplasm and large round to oval nuclei (H&E; magnification, ×100, scale bar: 100 μm). Obvious keratinization was detected focally (inset; H&E; magnification, ×400, scale bar: 20 μm). **D** Histopathological features of the GCLS component (accounting for 30%). It is composed of irregular sheets, trabeculae, ill-defined tubules or syncytia of polygonal cells embedded within prominent lymphocytic infiltrate (H&E; magnification, x100, scale bar:100 μm; inset, magnification, x400, scale bar: 20 μm). **E** AB-PAS staining shows positivity in AC component (H&E; magnification, ×100, scale bar: 100 μm) and AB-PAS-positive mucus is noted in the tubular glands (inset, magnification, x400, scale bar: 20 μm). **F** Diffuse positive for p63 (H&E; magnification, ×100, scale bar: 100 μm) and for p40 (inset, magnification, ×400, scale bar: 20 μm) in SCC. **G** Positive for CK5/6 in SCC (H&E; magnification, ×100, scale bar: 100 μm) and negative in GCLS (inset, magnification, ×400, scale bar: 20 μm). **H** EBER is detected in GCLS (H&E; magnification, ×100, scale bar: 100 μm) and in AC with glandular formation (inset, magnification, ×400, scale bar: 20 μm). **I** EBER is diffuse positive in SCC (H&E; magnification, ×100, scale bar: 100 μm; inset, magnification, ×400, scale bar: 20 μm)

**Fig. 2:**
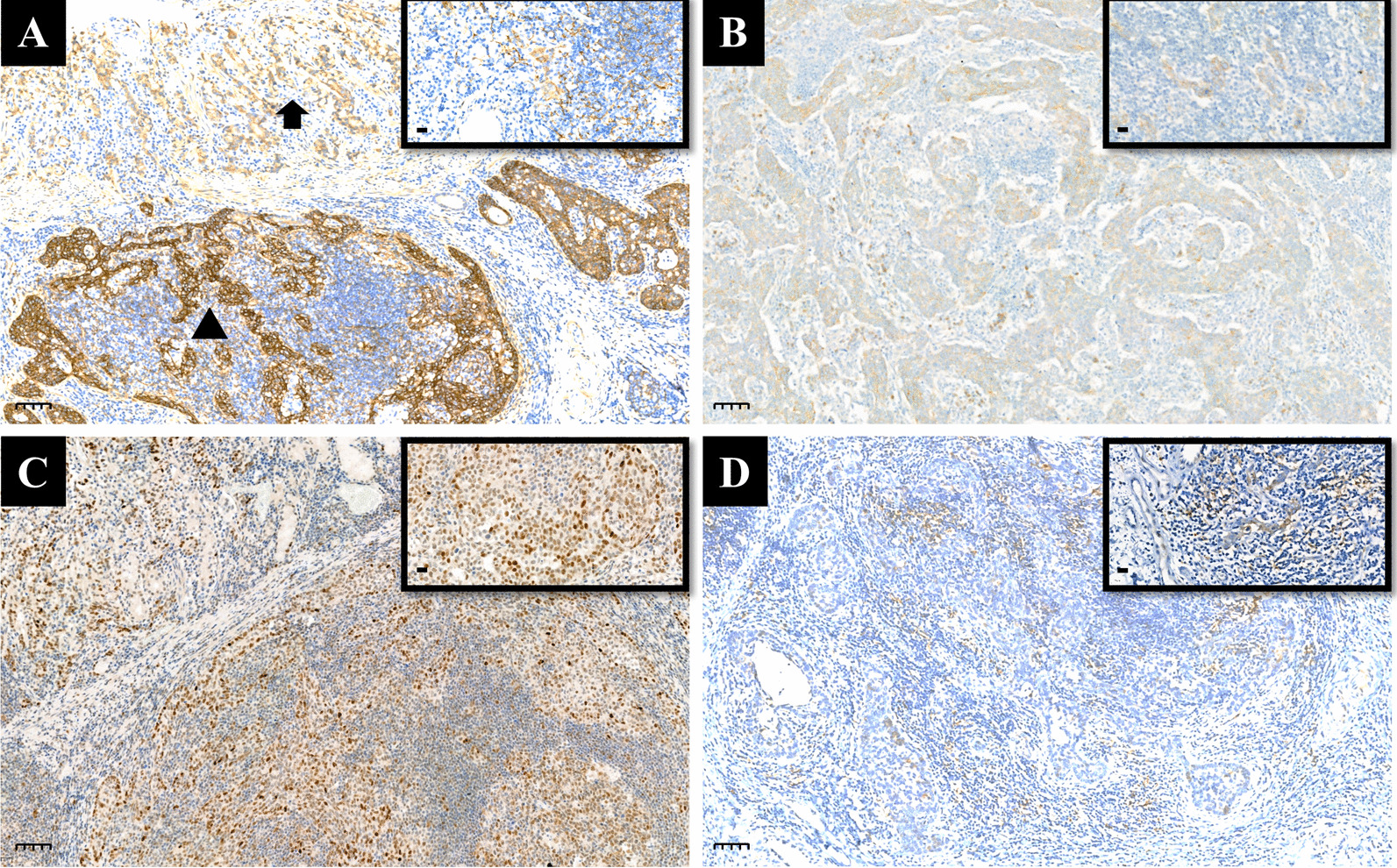
The expression of therapy-related proteins in the gastric tumors. **A** Strong and diffuse positive for EGFR in SCC (*Triangle*; magnification, ×100, scale bar: 100 μm), but weak to moderately positive in AC (*arrow*; magnification, ×100, scale bar: 100 μm) and GCLS (inset, magnification, ×400, scale bar: 20 μm). **B** Immunohistochemical score for HER2 expression is 1 + in SCC (magnification, ×100, scale bar: 100 μm) and GCLS (inset, magnification, ×400, scale bar: 20 μm). **C** p53 staining is patchy positive in SCC (magnification, ×100, scale bar: 100 μm; inset, magnification, ×400, scale bar: 20 μm). **D** Positive expression for PD-L1 on lymphocytes in and around SCC (magnification, ×100, scale bar: 100 μm) and GCLS (inset, magnification, ×400, scale bar: 20 μm). The combined positive score for PD-L1 expression is 15 (CPS = 15)

The endoscopic feature of the tumor was shown in Fig. 1A. Gross examination of the tumor showed that the tumor invaded the submucosa, so the tumor and the surrounding 2 cm gastric wall were continuously collected according to the sampling method of early gastric cancer. Histological examination revealed that the tumor was composed of a mixture of SCC (accounting for approximately 40%), poorly differentiated AC with intramucosal signet ring cell carcinoma (accounting for approximately 30%) and GCLS (accounting for approximately 30%) (Fig. 1B–D). The AC component had two morphologies. One was signet ring cell carcinoma, which was mainly located in the mucosal layer and was detected in the previous biopsy. The other was poorly differentiated tubular adenocarcinoma with focal glandular formation (Fig. 1B). SCC was arranged in nests, and the cells were polygonal with relatively rich eosinophilic cytoplasm. An intercellular bridge was noted, and obvious keratinization was detected focally (Fig. 1C). Lymphocyte infiltration was found between or around the AC and SCC, but not as much as in GCLS. GCLS was characterized by irregular trabeculae, sheets, poorly differentiated tubules or single polygonal cells embedded within abundant lymphocytes (Fig. 1D). All components invaded into the submucosa layer, and the deepest part of the invasion was about 5 mm from the mucosal muscularis. AB-PAS-positive mucus was noted in the signet ring cell carcinoma and poorly differentiated tubular AC (Fig. 1E), but not in SCC and GCLS. Immunohistochemically, p40, p63 and cytokeratin CK5/6 were diffusely positive in SCC (Fig. 1F, G), but were negative in AC and GCLS (Fig. 1F, G). CK7 was negative in all components, and mismatch repair proteins such as MLH1, PMS2, MSH2 and MSH6 remained intact. In situ hybridization detected EBER-positive neoplastic cells in all components (Fig. 1H, I). SCC, like AC and GCLS, showed diffuse positivity for EBER. EGFR was strongly positive in SCC, but weak to moderately expressed in AC and GCLS (Fig. 2A). Immunohistochemical score for HER2 expression was 1 + and p53 staining was patchy positive in all three components (Fig. 2B, C). Combined positive score (CPS) for PD-L1 expression was 15 (CPS = 15), because of positive expression on lymphocytes. Metastasis was found in one lymph node of group 4sa (1/54). The metastatic cells were in clusters without obvious glandular structure. They were negative for p40, p63 and CK7. AB-PAS staining showed positivity in individual cells, which indicated that the metastatic component was poorly differentiated AC.

Accordingly, a diagnosis of early EBV associated gastric ASC with concurrent GCLS (pT1bN1) was made. The patient took a single-drug S1 periodically for half a year after the surgery. The dosage was 60 mg/time, twice a day. The patient has been disease free during 8 months of medical follow-up.

## Discussion and conclusions

Gastric ASC with concurrent GCLS is extremely rare. To date, only three limited cases have been reported in the literature. EBV infection was positive in the concomitant GCLS, but none in the ASC. This case is the first case of gastric cancer with EBV infection detected in both ASC and GCLS. In addition, Ji-Hoon et al. reported a case of GCLS with focal SCC differentiation, which showed diffuse EBER positive in GCLS and focal EBER positive in SCC. The proportion of SCC in this case was 15%, which does not meet the diagnostic criteria for gastric ASC, that is, the proportion of SCC is greater than or equal to 25% [4]. The clinicopathological characteristics of these 4 cases and this present one are summarized in Table 1.

**Table 1:** Clinicopathological features of gastric cancer with two components of adenosquamous carcinoma and gastric carcinoma with lymphoid stroma

Cases	Age/sex	Location	Size mm, (Gross type)	p63/p40	EBER	Metastatic components	TNM Stage	Prognosis
Case 1	67/male	Upper	One lesion 60 × 50(Type III)	AC(−) SCC(+) GCLS(−)	AC(−) SCC(−) GCLS(+)	NA	NA	NA
Case 2	50/male	Angle	One lesion 90 × 85 (type II)	Not done	AC(−) SCC (−) GCLS (+)	NA	NA	AWD, 10 years
Case 3	58/male	Antrum	Two lesions 35 × 25 (ASC, Type III) 45 × 36 (GCLS, Type III)	AC(−) SCC(+) GCLS(−)	AC(−) SCC(−) GCLS (+)	GCLS	T4aN2	AWD, 8 months
Case 4	58/male	Angle and body	Two lesions 11 × 11 (GCLS with focal SCC, type IIb) 30 × 20 (GCLS, type IIc)	GCLS(−) SCC(+)	SCC(focal +) GCLS(+)	GCLS	T1bN3a	Recurrence in 12 months and death in 25 months
Present case	59/male	Body	One lesion 30 × 30 (GSC + GCLS, type IIc)	AC(−) SCC(+) GCLS(−)	AC(+) SCC( diffuse +) GCLS(+)	AC	T1bN1	AWD in 8 months

*NA* not available, *AC* adenocarcinoma, *GCLS* gastric carcinoma with lymphoid stroma, *SCC* squamous cell carcinoma, *AWD* alive without disease, *EBER* Epstein‑Barr virus encoding RNA

The pathogenesis of gastric ASC has been under debate. There are several hypotheses proposed: (i) collision of concurrent AC and SCC, (ii) originating from the same cancer stem cell, which can differentiate into AC and SCC, (iii) oncogenic transformation of metaplastic squamous cells, (iv) squamous metaplastic transformation from the existing AC or GCLS. Considering that there was a transition between AC and SCC in the present case, and AC was the most frequent component in the metastatic lymph nodes with gastric ASC as indicated in the literature and also in our case [6, 7], we suggest the last possibility.

The role of EBV in gastric SCC has been unclear. Takita et al. detected EBV infection in the surgical specimens of gastric SCC by polymerase chain reaction [8]. In the present case, SCC component showed diffusely positive for EBER in ISH assay. These findings suggest that EBV infection may play an important role in the pathogenesis of some gastric SCC. We speculate that EBV infection is a relatively early molecular event in the development of gastric SCC, and the molecular mechanism needs to be further studied.

In our case, there was significant difference in EGFR expression between the two components of AC and SCC, but not in other proteins such as HER2, p53 and mismatch repair proteins. The role of EGFR pathway in the differentiation of SCC in gastric ASC is worthy of further exploration. There were abundant lymphocytes infiltrating around and inside the tumor. These cells highly expressed PD-L1, and the CPS of the tumor was 15, interpreted as positive. A high correlation between EBV infection and PD-L1 expression has been discovered, suggesting patients with EBV-positive gastric cancer may benefit from immunotherapy [9].

We reported a rare case of gastric ASC with concurrent GCLS, with EBV infection detected in both ASC and GCLS. Pathologists and clinicians should recognize and pay attention to such a tumor. Further studies are needed to explore the pathogenesis and biological behavior of this type of tumor.

## Data Availability

All data analyzed are included in the published article.

## Abbreviations

EBV: Epstein–Barr virus
EBV-GC: Epstein–Barr virus-associated gastric cancer
EBER: EBV-encoded RNA
GCLS: Gastric carcinoma with lymphoid stroma
ASC: Adenosquamous carcinoma
SCC: Squamous cell carcinoma
AC: Adenocarcinoma
WHO: World Health Organization
EGFR: Epidermal growth factor receptor
HER2: Human epidermal growth factor receptor 2
AB-PAS: Alcian-blue periodic-acid-schiff stain.
PD-L1: Programmed death ligand 1
CPS: Combined positive score

## References

[1] Ignatova E, Seriak D, Fedyanin M (2020). Epstein–Barr virus-associated gastric cancer: disease that requires special approach. Gastric Cancer.

[2] Pyo JS, Kim NY, Son BK (2020). Clinicopathological features and prognostic implication of gastric carcinoma with lymphoid stroma. Gastroenterol Res Pract.

[3] Feng F, Zheng G, Qi J (2017). Clinicopathological features and prognosis of gastric adenosquamous carcinoma. Sci Rep..

[4] Nagtegaal ID, Odze RD, Klimstra D (2020). The 2019 WHO classification of tumours of the digestive system. Histopathology.

[5] Li HS, Liu X, Zhang MY (2020). Clinicopathologic characteristics, survival, and treatments for gastric adenosquamous carcinoma: a population-based study. Curr Oncol.

[6] Ji-Hoon K, Dae-Woon E (2019). A case of multifocal EBV associated gastric carcinoma with focal squamous differentiation. Indian J Pathol Microbiol.

[7] Miyake H, Miyasaka C, Ishida M (2019). Simultaneous gastric adenosquamous carcinoma and gastric carcinoma with lymphoid stroma: a case report. Mol Clin Oncol.

[8] Takita J, Kato H, Miyazaki T (2005). Primary squamous cell carcinoma of the stomach: a case report with immunohistochemical and molecular biologic studies. Hepatogastroenterology.

[9] Xie T, Liu Y, Zhang Z (2020). Positive status of Epstein–Barr virus as a biomarker for gastric cancer immunotherapy: a prospective observational study. J Immunotherapy.

